# A Web-Based Study of Dog Ownership and Depression Among People Living With HIV

**DOI:** 10.2196/mental.8180

**Published:** 2017-11-08

**Authors:** Abigail L Muldoon, Lisa M Kuhns, Julie Supple, Kristen C Jacobson, Robert Garofalo

**Affiliations:** ^1^ Division of Adolescent Medicine Ann & Robert H Lurie Children’s Hospital of Chicago Chicago, IL United States; ^2^ Department of Pediatrics Feinberg School of Medicine Northwestern University Chicago, IL United States; ^3^ Test Positive Aware Network Chicago, IL United States; ^4^ Department of Psychiatry & Behavioral Neuroscience The University of Chicago Chicago, IL United States

**Keywords:** HIV, depression, pet-human bonding

## Abstract

**Background:**

People living with human immunodeficiency virus (PLHIV) are approximately twice as likely to be depressed compared with HIV-negative individuals. Depression is consistently associated with low antiretroviral therapy (ART) adherence, an important step within the HIV care continuum related to HIV disease progression and overall health. One factor that may have positive psychosocial benefits and promote ART adherence is dog ownership. Research indicates that dog ownership is associated with lower depression, and initial evidence suggests its positive impact on psychosocial outcomes for PLHIV.

**Objective:**

The aim of our study was to expand the existing research by examining the relationship between current dog ownership and depression for a sample of PLHIV while controlling for demographic characteristics and other potential confounders.

**Methods:**

Participants aged 18 years or older and who self-reported an HIV diagnosis were recruited via social media into *When Dogs Heal*, a cross-sectional Web-based survey to collect data among adult PLHIV. The research visit was conducted via a Web-based survey, and there was no in-person interaction with the participant. Primary outcome measures included demographic questions (age, race, ethnicity, gender, and sexual orientation), pet ownership (type of pet owned and current dog ownership), depression (Center for Epidemiologic Studies Depression Scale, 10 items), and resilience (Resilience Research Centre Adult Resilience Measure, 28 items).

**Results:**

A total of 252 participants were enrolled into the study in January 2016, with a final analytic sample of 199 participants. Mean age was 49 years, 86.4% (172/199) of participants were male, and 80.4% (160/199) were white. Current dog ownership was prevalent among the sample (68.3%, 136/199). Bivariate analysis indicated that there was no significant relationship between depression and demographic characteristics (age, race, ethnicity, gender, and sexual orientation), with *P*>.05. The multivariate logistic regression, including age, race, ethnicity, gender, resilience, and current dog ownership, was significant, with *P*<.001. Of the 6 predictor variables, only 2 were statistically significant: dog ownership and resilience. Noncurrent dog owners had 3 times higher odds of depression in comparison with current dog owners: odds ratio 3.01; 95% CI 1.54-6.21.

**Conclusions:**

Growing evidence suggests that dog ownership reduces the likelihood of depression and, therefore, may confer long-term health benefits on PLHIV. Future studies should explore whether dog-specific interventions are a feasible and efficacious intervention to improve outcomes among PLHIV.

## Introduction

In the United States, 1.1 million people are living with human immunodeficiency virus (HIV), with the highest burden among men who have sex with men (MSM), particularly racial and ethnic minority MSM [1]. The improved life expectancy of individuals living with HIV, largely because of antiretroviral therapy (ART) [2] and the shift to treatment as prevention [3], has increased the focus in the United States on linkage to care, engagement, and retention in medical care (including medication adherence), and viral suppression across the HIV care continuum. Among people living with HIV (PLHIV), a high level of ART adherence (at least 80%) may be needed to suppress viral replication and avoid resistance [4-6]; suboptimal adherence can lead to disease progression and decreased life expectancy for HIV-related disease [7-9]. Although the goal in the United States is suppression of the virus in 80% of infected individuals, in the most recent analysis, only 55% of PLHIV were virally suppressed [3,10].

Depression and depressive symptoms are consistently associated with poor ART adherence and overall HIV disease progression—even in a post-ART era [11-15]. Notably, depressive symptoms are prevalent among PLHIV [16-18]. Although it is estimated that approximately 16.1% of all Americans have been diagnosed with depression in their lifetime [19], one large national study (N=1560) found that 63% of PLHIV have experienced depressive symptoms (ie, depressive symptoms lasting 2 weeks or longer in their lifetime) [20]. In addition, Ciesla and Roberts’s meta-analysis revealed that PLHIV are almost 2 times more likely to have had a recent major depressive episode (as measured by a diagnostic clinical interview) compared with HIV-negative individuals [21]. Interventions targeting depressive symptoms, particularly mild to moderate symptoms, are warranted and may help to improve HIV-specific health outcomes more generally [22].

One factor that may ameliorate depressive symptoms, promote ART adherence, and potentially assist in improving other HIV-related health outcomes is pet ownership. Pet ownership, in particular dog ownership, has been linked to multiple positive physical, psychological, and psychosocial health outcomes [23]. Evidence from studies exploring pet ownership and other animal-assisted interventions (AAIs) suggest positive physical health outcomes include cardiovascular benefits [24-26], increased physical activity [27-31], reduced cortisol levels [32], and lower blood pressure [33,34], particularly in response to stress [24,32,33,35,36]. Dog ownership is also associated with improved psychological outcomes, including lower depression [37-39], decreased loneliness [30,38,40], and improved general psychological well-being [29,38]. Additionally, evidence suggests that pet dogs facilitate social interactions [30,41-43], act as a catalyst in building social networks [30,43,44], and act as a conduit for building social capital [30,45].

Dog-specific AAIs have been shown to benefit those living with chronic health conditions in particular. Hospitalized pediatric cancer patients who undergo animal-facilitated therapy with dogs show improved psychological health, decreased worry, and less fear [46,47]. In a review of related literature, Urbanski and Lazenby concluded that, overall, animal-facilitated therapy benefits children living with chronic illnesses, including those who are immunocompromised [48]. An increasing number of people with diabetes are acquiring diabetic alert dogs (DADs) for hypoglycemia detection. Although the evidence demonstrating DADs’ ability to accurately detect hypoglycemia is limited and inconclusive, studies indicate that there are positive psychosocial outcomes, including decreased worry [49,50]. The first study to evaluate the objective reliability of DADs in a real-world environment found that despite the 12% positive predictive value, using DADs was feasible and participants were very satisfied with their dog [51]. One vulnerable population with a long-term health condition that may benefit from AAIs with pet dogs is PLHIV.

Similar to the general population, a small body of research suggests positive benefits of pet ownership among PLHIV, including stress reduction and improvement in mood and well-being, particularly for those with less social support [52-56]. One of the first studies to examine the impact of companion animals on PLHIV was a small descriptive study from 1991 [52]. Drawing themes from interviews with a small sample of gay men with HIV/AIDS (acquired immune deficiency syndrome), companion animals were found to provide social support, a means to reduce stress, and a sense of purpose because of the continuous care that participants needed to provide to their pet [52].

The first methodologically rigorous investigation into pet ownership in a sample of gay and bisexual men used data from the Multicenter AIDS Cohort Study, an ongoing observational study of the natural history of HIV/AIDS [57]. Siegel et al analyzed data from 1872 participants of which 708 (38%) were HIV-positive and 214 (11%) had AIDS [55]. For those diagnosed with AIDS, having a pet was associated with less depression, especially among those with lower social support [55]. More recently, a study conducted in 2011 with male US military veterans living with HIV/AIDS found dog ownership positively influenced well-being through companionship, sense of responsibility, and stress reduction [53]. Another cross-sectional study conducted in 2011 among 128 PLHIV in Australia (92% male, 87% nonheterosexual) found that those living with a companion animal (the most common was a dog) had higher levels of emotional well-being and decreased HIV-related negative social interactions compared with those without a companion animal [56].

In sum, initial evidence suggests that dog ownership is associated with positive psychosocial outcomes for PLHIV. In the study described herein, we sought to update this evidence and extend it to a broad target population of PLHIV in the United States. On the basis of prior findings, we hypothesized that among PLHIV, current dog ownership would be associated with fewer depressive symptoms, controlling for demographic characteristics and other potential confounders. Given the potential for dog ownership to reflect a resilient response to stress (eg, drawing on social capital and social interaction), we also measured and included an indicator of resilience in our models to control for its potential confounding of the dog ownership and depression relationship.

## Methods

### Participants and Procedure

The study *When Dogs Heal* utilized an open cross-sectional Web-based survey to collect data among adult PLHIV. To recruit participants, messages were posted to social media platforms (eg, Facebook and Twitter) by the principal investigator, who posted about the study one time on each platform. Participants were incentivized to participate by offering a chance to win an Apple iPad. Eligibility criteria included was being ≥18 years old and self-reporting an HIV diagnosis; dog ownership was not a requirement to enroll. Interested individuals were directed to a Web link with the study screener, consent statement, and survey. The survey was thoroughly tested by study staff before data collection. Upon clicking the link, volunteers were instructed to complete a series of screening questions and, if eligible, proceeded through the consent process before completing the study questionnaire. The survey questionnaire was deployed using Qualtrics, a Web-based data collection software tool based out of Seattle, WA and Provo, UT. Qualtrics utilizes transport layer security (TLS) encryption for all transmitted data and services are hosted by secure data centers. Participants were able to review and change their previous answers through a back button and were typically shown 1 question per page. Browser cookies and Internal Protocol (IP) address were used to prevent duplicate responses from the same individual. This study was approved by the institutional review board at the study site.

### Measures

Participants first completed a brief set of demographic questions indicating their primary race (Asian/Pacific Islander, black/African American, white, Latino/Hispanic, other, or multiracial), ethnicity (Spanish/Hispanic/Latino/a), sex assigned at birth (male or female), current gender identity (male, female, or transgender), age, and whether or not they ever owned a pet (yes or no). Participants who reported having ever owned a dog were asked whether they currently owned a dog.

Depression was measured using the shortened Center for Epidemiologic Studies Depression Scale (CES-D10) [58,59].This 10-item self-report measure of depressive symptoms in the prior week uses a 4-point frequency response scale. This measure has demonstrated excellent reliability and validity in prior research [58,60], as well as in this study (alpha=.90). The CES-D10 was scored by summing the 10 items, with higher scores indicating increased depressive symptoms. Per scoring criteria, a cut-off score of 10 or greater was used to determine whether the participant met the criteria for depression.

Resiliency, a potential confounding variable, was measured with the Resilience Research Centre Adult Resilience Measure (RRC-ARM), an adapted version of the Child and Youth Resilience Measure (CYRM-28) [61,62]. The 28-item instrument measures the resources available to individuals that may boost their ability to sustain their well-being through individual resources or capabilities, relationship with primary caregivers, and contextual factors that facilitate a sense of belonging [62]. Cronbach alpha for the 3 subscales of the CYRM-28 range from .794 to .833 and subscale correlations range from .555 to .705 [63]. This sample yielded an alpha of .90. An overall resiliency score was created by averaging across all items to achieve a score 1-5, where higher scores indicate higher levels of resilience.

### Data Analysis

We used descriptive statistics, including measures of central tendency and dispersion, to characterize the sample. We assessed simple associations between variables using Pearson correlations or chi-square tests, with significance level set at alpha <.05. A multivariable logistic regression analysis assessed the log odds that a participant would meet criteria for depression based on current dog ownership, controlling for age, race, ethnicity, and gender, as well as resilience. Due to low cell numbers, the race variable was dichotomized for analysis (white vs nonwhite). Data were analyzed using SPSS statistics version 24 (IBM Corporation).

## Results

### Participant Characteristics

In total, 356 data cases were downloaded from Qualtrics, of which 29 cases had a duplicate IP address. The data cases that were most complete were kept in the final dataset, leaving 327 unique volunteers who clicked the survey link; 15 cases that contained no data were deleted (ie, a volunteer clicked the link but did not proceed past the first screen). A total of 28 cases were deleted for not meeting eligibility criteria. Of the participants who were eligible, 32 did not enroll in the study.

A total of 252 adults living with HIV were enrolled into the study in January 2016, yielding a participation rate of 77.1% (252/327). Completion of the study survey took approximately 15 min on average. A total of 51 cases were withdrawn from analysis for largely incomplete responses. The completion rate for the survey was 79.8% (201/252). An additional 2 participants who reported transgender status were removed from the sample because of small cell size for analysis, that is, to minimize the number of variables in analytical models. The final analytic sample included 199 PLHIV.

**Table 1:** Sample characteristics of adult people living with human immunodeficiency virus (N=199).

Characteristic			Number
Age at baseline, mean (SD)			48.72 (10.68)
Age at HIV diagnosis, mean (SD)			31.89 (9.43)
Years since human immunodeficiency virus (HIV) diagnosis^a^, mean (SD)			16.87 (10.05)
**Gender**			
	Male, n (%)		172 (86.4)
	Female, n (%)		27 (13.6)
**Race**			
	Black/African American, n (%)		9 (4.5)
	White, n (%)		160 (80.4)
	Latino/Hispanic, n (%)		9 (4.5)
	Asian/Pacific Islander, n (%)		7 (3.5)
	Other and multiracial, n (%)		14 (7.0)
**Ethnicity**			
	Hispanic/Spanish/Latino/a, n (%)		18 (9.0)
	Not Hispanic/Spanish/Latino/a, n (%)		181 (91.0)
**Sexual orientation**			
	Homosexual/gay, n (%)		165 (82.9)
	Bisexual, n (%)		8 (4.0)
	Heterosexual/straight, n (%)		23 (11.6)
	Other, n (%)		3 (1.5)
**Ever owned a pet**			
	Yes, n (%)		194 (97.5)
	No, n (%)		5 (2.5)
**Ever owned a dog**			
	Yes, n (%)		184 (92.5)
	No, n (%)		15 (7.5)
	**Acquired dog before or after HIV diagnosis^a^**		
		Before, n (%)	57 (31.1)
		After, n (%)	126 (68.9)
	**Dog type**		
		Companion or household pet, n (%)	168 (91.3)
		Therapy dog, n (%)	14 (7.6)
		Official service dog, n (%)	2 (1.1)
**Current dog ownership**			
	Yes, n (%)		136 (68.3)
	No, n (%)		63 (31.7)

^a^One participant declined to answer this question.

Table 1 summarizes the characteristics of the sample. The mean age was 48.72 years (standard deviation, SD 10.68; range 21-73) with the mean age at HIV diagnosis of 31.89 years (SD 9.43; range 15-67). The average number of years since HIV diagnosis was 16.87 (SD 10.05; range 0-38). Most participants were male (86.4%, 172/199), white (80.4%, 160/199), not Hispanic/Spanish/Latino/a (91.0%, 181/199), and homosexual or gay (82.9%, 165/199). The vast majority of the sample reported lifetime pet ownership (97.5%, 194/199), with the most commonly reported pet being a dog (92.5%, 184/199). Of the participants who reported ever having owned a dog, 68.9% (126/183) reported they got their dog after their HIV diagnosis, and 91.3% (168/184) indicated it was a household pet as compared with a therapy dog or official service dog. Current dog ownership was prevalent among the sample (68.3%, 136/199).

### Bivariate Analysis

Chi-square tests (with Fisher exact test as needed for low cell sizes) and Pearson correlations indicated that there was no significant relationship between depression and demographic characteristics (N=199): age (*r*=−.08, *P*=.28), race (χ^2^_1_=1.8, *P*=.21), ethnicity (χ^2^_1_<0.1, *P*>.99), gender (χ^2^_1_=0.5, *P*=.54), and sexual orientation (χ^2^_1_=0.6, *P*=.51). Resilience was moderately and negatively correlated with depression (*r*=−0.31, *P*<.001) (data not shown).

### Multivariate Regression

Table 2 presents the results of the multivariable logistic regression model. Although not significant in bivariate analysis, we retained age, race, ethnicity, and gender in the model for substantive reasons. The logistic regression model was statistically significant, χ^2^_6_=37.206, *P*<.001. Of the 6 predictor variables, only 2 were statistically significant: dog ownership and resilience. Noncurrent dog owners had 3 times higher odds of depression in comparison with current dog owners (OR 3.01, 95% CI 1.54-6.21), controlling for demographic factors and resilience.

**Table 2:** Multivariable logistic regression of depression on pet ownership among people living with human immunodeficiency virus (N=199).

Predictor Variables	Beta	Standard error	*P* value	Odds ratio (95% CI)
Age	−.026	0.015	.08	0.974 (0.946-1.004)
Race (white)	−.687	0.460	.14	0.503 (0.204-1.239)
Ethnicity (Hispanic)	−.203	0.605	.74	0.816 (0.249-2.671)
Gender (male)	−.508	0.500	.31	0.602 (0.226-1.602)
Resilience	−1.201	0.291	<.001	0.301 (0.170-0.533)
Current dog ownership (no)	1.130	0.355	.001	3.095 (1.542-6.213)
Constant	6.427	1.622	<.001	618.620

## Discussion

### Principal Findings

This study explored the impact of current dog ownership on depression for a sample of adult PLHIV. It was hypothesized that among PLHIV, current dog ownership would be associated with decreased likelihood of meeting depressive criteria. Our hypothesis was supported in that noncurrent dog ownership was significantly and positively associated with depression, controlling for the potential influence of resilience and other demographic factors. Individuals who were not current dog owners had 3 times higher odds of depression compared with current dog owners. This finding adds to and updates prior findings regarding the potential positive impact of dog ownership on depression in PLHIV.

This is a notable finding as depression is the most common psychiatric disorder associated with HIV disease [64]. Depression influences not only the psychological health of PLHIV but is a correlate of overall HIV disease progression as well. Support for or promotion of dog adoption and ownership may be a novel intervention to positively impact depression and, in turn, positively affect other HIV-related health outcomes. The growing adoption of dogs for diabetes-specific intervention and related research will inform feasibility and potential efficacy for PLHIV. The high satisfaction reported by dog owners in DAD programs provides preliminary evidence that full-time dog adoption may be feasible for other populations with chronic illnesses, such as PLHIV. Although research to date suggests benefits of dog ownership among PLHIV, longitudinal and intervention research is needed to establish whether or not dog adoption is effective in reducing psychological symptoms and promoting overall health.

Although little research has been done on the potential mechanisms of action, dog ownership may impact depression and overall psychological health among PLHIV through bolstering social support networks. Pet dogs may act as a source of social support that can supplement existing support networks or fill network voids for vulnerable populations [24,30,35-39,44,52,65]. Allen and colleagues found evidence that pets, specifically dogs, act as sources of nonevaluative social support when faced with a stressor. This nonevaluative social support may facilitate better and faster physiological responses to mental stress over and above spousal and friend support [24,35,36]. The numerous positive associations of social support for PLHIV, including reduced depression, have been well documented [66,67]. Dog ownership may provide a unique type of social support for PLHIV that positively impacts depression over and above their existing social support networks.

Beyond ameliorating depressive symptoms, dog ownership may also have an effect on other important factors related to health outcomes for PLHIV, including ART adherence. As noted earlier, depression is consistently associated with suboptimal ART adherence. Inadequate social support is also routinely found to be correlated with poor ART adherence [68,69]. One additional mechanism through which dog ownership may influence ART adherence is through the routinization associated with pet care, which reflects the extent to which one’s life is organized and follows a predictable pattern [52-54,70]. Routinization has been found to be an important factor in facilitating ART adherence [71-75]. Preliminary evidence suggests that pets may facilitate the adoption of daily routines into which ART regimens can be incorporated [52-56]. Daily and continuous care for the dog through walking and feeding can provide a source of social interaction and give PLHIV a sense of purpose while establishing a healthy routine for themselves. One qualitative study on routinization found that even when participants were experiencing homelessness, having at least one recurring daily activity was associated with >70% adherence to ART; among the daily activities discussed was walking their dog [56]. Although ART adherence and routinization were not measured in this study, future studies should explore these factors, in addition to social support, as potential mediators of dog ownership on health outcomes for PLHIV.

### Limitations

Several limitations of this study should be noted. The data are cross-sectional in nature and therefore causality cannot be inferred. For example, we measured depressive symptoms over the past 7 days, meaning we do not have data regarding onset of depression or whether depressive symptoms preceded or followed dog companionship. Additionally, only participants with access to the Internet were able to enroll. Although the vast majority of Americans report having access to the Internet, Internet adoption is still closely dependent on income and education [76]. This sample comprised mostly middle-aged, nonheterosexual white men who had been infected for more than 10 years (on average), which is not reflective of the current trends in HIV incidence among younger racial and ethnic minorities. Therefore, findings may not generalize to younger and recently infected PLHIV.

### Conclusions

In conclusion, growing evidence suggests that dog ownership reduces the likelihood of depression and, therefore, may confer long-term health benefits on PLHIV. Given this evidence, testing of dog-specific AAIs to reduce depressive symptoms as well as to improve HIV-related health outcomes is a logical next step. Given the relatively low rate of viral suppression in PLHIV in comparison with national targets, innovative interventions are needed to reach goals established under the national HIV/AIDS strategy.

## Abbreviations

AAI: animal-assisted intervention
AIDS: acquired immune deficiency syndrome
ART: antiretroviral therapy
CES: Center for Epidemiologic Studies
DAD: diabetic alert dog
HIV: human immunodeficiency virus
IP: Internet Protocol
MSM: men who have sex with men
PLHIV: people living with HIV
RRC: Resilience Research Centre
TLS: transport layer security
